# Developmental differences in masked form priming are not driven by vocabulary growth

**DOI:** 10.3389/fpsyg.2014.00667

**Published:** 2014-07-11

**Authors:** Adeetee Bhide, Bradley L. Schlaggar, Kelly Anne Barnes

**Affiliations:** ^1^Department of Neurology, Washington University School of MedicineSt. Louis, MO, USA; ^2^Department of Radiology, Washington University School of MedicineSt. Louis, MO, USA; ^3^Department of Pediatrics, Washington University School of MedicineSt. Louis, MO, USA; ^4^Department of Anatomy and Neurobiology, Washington University School of MedicineSt. Louis, MO, USA

**Keywords:** priming, vocabulary, lexical precision, reading, developmental

## Abstract

As children develop into skilled readers, they are able to more quickly and accurately distinguish between words with similar visual forms (i.e., they develop precise lexical representations). The masked form priming lexical decision task is used to test the precision of lexical representations. In this paradigm, a prime (which differs by one letter from the target) is briefly flashed before the target is presented. Participants make a lexical decision to the target. Primes can facilitate reaction time by partially activating the lexical entry for the target. If a prime is unable to facilitate reaction time, it is assumed that participants have a precise orthographic representation of the target and thus the prime is not a close enough match to activate its lexical entry. Previous developmental work has shown that children and adults' lexical decision times are facilitated by form primes preceding words from small neighborhoods (i.e., very few words can be formed by changing one letter in the original word; low N words), but only children are facilitated by form primes preceding words from large neighborhoods (high N words). It has been hypothesized that written vocabulary growth drives the increase in the precision of the orthographic representations; children may not know all of the neighbors of the high N words, making the words effectively low N for them. We tested this hypothesis by (1) equating the effective orthographic neighborhood size of the targets for children and adults and (2) testing whether age or vocabulary size was a better predictor of the extent of form priming. We found priming differences even when controlling for effective neighborhood size. Furthermore, age was a better predictor of form priming effects than was vocabulary size. Our findings provide no support for the hypothesis that growth in written vocabulary size gives rise to more precise lexical representations. We propose that the development of spelling ability may be a more important factor.

## Introduction

Learning to read, unlike learning to speak, requires explicit instruction. Models of reading attempt to account for reading performance across development. However, several unresolved questions prevent models of reading skill acquisition from being further refined. Specifically, what are the mechanisms that allow fluent readers to distinguish between words that are visually similar but have different meanings?

Masked priming paradigms (Forster et al., 1987) provide a means for studying developmental changes in orthographic processing. In masked priming paradigms, a prime is presented briefly (c. 50 ms) and is masked by a row of hash marks that precedes it and a target word that follows it (typically in a different-case font). Participants are typically unaware of the primes because of their rapid and masked presentation, and hence cannot use different strategies for processing the primes. Therefore, this paradigm is particularly useful for studying developmental changes because it can distinguish age-related differences from differences in strategic processing.

Form priming, where the prime and target differ by a single letter (e.g., clee-FLEE), provides a way to measure the precision of orthographic representations. Form priming in adults varies as a function of orthographic neighborhood size (N), the number of words that can be formed from a target word by changing a single letter. For example, the word *echo* has no orthographic neighbors, whereas the word *yell* has 9 orthographic neighbors including *yelp* and *cell*. For adults, masked non-word form primes significantly facilitate lexical decision times relative to unrelated primes (e.g., pilk-FLEE) when the target word has few orthographic neighbors, but not when the target word has many neighbors (Forster, 1987; Segui and Grainger, 1990; Forster and Davis, 1991; Castles et al., 1999).

Competing hypotheses have been proposed regarding the mechanisms underlying neighborhood size effects on adult form priming. The first hypothesis, an entry-opening search model (Forster and Davis, 1984; Forster, 1989), is predicated on the idea that word detectors are more sharply tuned for high than low N words, to minimize confusion with other visually similar words. This sharper tuning may be accomplished by recoding words into their bodies and antibodies (Forster and Taft, 1994). According to this hypothesis, form primes are not sufficiently close matches to high N words to facilitate response times, resulting in less priming for high than low N words. Another hypothesis, a network framework model based on Rumelhart and McClelland's (1982) interactive activation model, posits that greater levels of competition or inhibition between neighbors for high than low N targets offset the facilitatory effect of the prime. A recent study from Andrews and Hersch (2010) found that for adults who were “above average” spellers, form primes preceding high N targets led to a significant slowing of lexical decision response time (i.e., the prime inhibited rather than facilitated response times). Such inhibitory effects are not reconcilable with search models, but can be accommodated by models postulating inhibitory links.

Masked form priming for high N words has been shown to vary across development, suggesting experience, maturation, or an interaction between the two alters the mechanisms that yield this behavioral effect. Unlike adults, children show masked form priming for both low and high N words (Castles et al., 1999, 2003, 2007). Castles et al. (1999) initially hypothesized that written vocabulary growth, and its effects on either lexical tuning or lexical competition (Castles et al., 2007), is related to developmental differences in neighborhood density effects on form priming. Since children have smaller written vocabularies than adults, they may not know all of the neighbors of a high N target, rendering the target effectively low N. As neighbors of a particular word are learnt, vocabulary growth would either initiate the recoding of high N words or increase the number of inhibitory links associated with that particular word. Some studies support this theory, documenting attenuation of form priming with age (Castles et al., 2007). Other studies have continued to show large priming effects during developmental periods when written vocabulary was presumed to increase. For example, Castles et al. (1999) reported that children continued to show facilitation from 2nd grade until 6th grade, when vocabulary testing revealed that the high N targets were effectively high N for the 6th graders. This finding led to the hypothesis that a neighborhood density threshold has to be reached before lexical detectors begin to narrow their tuning. This hypothesis was supported by the observation that the 6th graders with the highest sight vocabularies showed no form priming for high N targets (Castles et al., 1999).

The purpose of this paper is to test the hypothesis that differences in written vocabulary size underlie developmental differences in form priming. To test this hypothesis, we controlled for, and quantified, effective N. We used very low N (0–1 neighbors) and very high N (≥10 neighbors) targets, which afforded us two potential advantages. First, the use of very high N stimuli provided an opportunity to test whether we could replicate Andrews and Hersch's (2010) finding of significant inhibition for high N form priming in adults, as such high N stimuli would be expected to generate substantial lexical competition. Second, the use of very high N stimuli increased the likelihood that these stimuli would be high N for young children, who might know only a fraction of the neighbors. We measured children's knowledge of these neighbors to determine whether individual differences in form priming related to individual differences in neighbor knowledge, as would be expected if vocabulary size related to high N form priming. As a final test, we created a “matched” set of stimuli whose average N equaled the estimated effective N for the youngest children. These matched stimuli were shown only to the adults to control for potential differences in effective N between children and adults. If significant differences were seen between the matched N stimuli in adults and the high N stimuli in children, then the likelihood is markedly reduced that vocabulary differences are the cause of developmental differences in priming. In the first analysis, we used linear mixed effects modeling to calculate the expected reaction time to stimuli that were preceded by different prime types as a function of age. We expected everyone to show facilitation due to repetition primes (e.g., flee-FLEE) and form primes preceding low N targets. However, we expected only the younger participants to show facilitation when form primes preceded high N targets. The second analysis also entailed a linear mixed effects model, but the matched N targets were inputted for the adults to control for effective neighborhood size. If we found no differences between the adults' matched N words and the children's high N words, then the results would support the written vocabulary growth hypothesis. In contrast, if we found significant differences between the two age groups, wherein children were facilitated in the high N form priming condition but adults were not facilitated in the matched N form priming condition, then the results would not support the written vocabulary growth hypothesis. In our final analysis, we tested whether age or vocabulary size was a better predictor of high N form priming effects in children and adolescents. Vocabulary being the better predictor would support the written vocabulary growth hypothesis, whereas the opposite result would not.

A second goal of this study was to map the developmental trajectory of form priming. Previous studies have primarily examined discrete age groups (e.g., testing children in 2nd, 4th, and 6th grade, as well as adults). This study is the first priming study, to our knowledge, to test age as a continuous variable from childhood through adulthood.

## Methods

### Participants

Twenty-seven adults (18–27 years old, 11 males), 26 adolescents (13–17 years old, 13 males), and 38 children (7–12 years old, 15 males) were recruited from Washington University in St. Louis and the St. Louis metropolitan area. All participants were monolingual, native English speakers, with normal or corrected-to-normal vision and no history of neurological or psychiatric disorders. Adult participants and parents of child and adolescent participants provided informed consent and child and adolescent participants provided informed assent. Participants were compensated $15/h. All aspects of the study were performed with the approval of the Washington University Human Studies Committee.

Subject data were included in the study if: (1) accuracy in each condition of the lexical decision task was above chance (50%), and (2) the participant did not report being able to read the primes. Thirteen children and 2 adolescents did not meet the accuracy criteria. One adult reported being able to see the primes. The final sample comprised 26 adults (18–23 years old, 11 males), 24 adolescents (13–17 years old, 13 males), and 25 children (8–12 years old, 12 males). Although we did not collect IQ and reading measures from all children, the available IQ and reading data did not differ between included and excluded participants. Additional demographic and psychometric data are reported in Table 1.

**Table 1:** **Participant information**.

	***N***	**Age in years**	***N***	**Age in years**	**IQ**	**Reading standard score**
Children	**38**	**10.31 (1.39)**	25	10.57 (1.14)	124^*^	118.62^*^
Adolescents	**26**	**15.10 (1.24)**	24	15.22 (1.20)	111	103.25
Adults	**27**	**20.98 (1.21)**	26	20.91 (1.18)	126^*^	N/A

Bold values pertain to all tested participants. Non-bold data pertain to the participants who met the inclusion criteria. The asterisk (

* *) signifies that the data is an estimate based on a subset of the included population. The Vocabulary and Matrix Reasoning Subtests of the Wechsler Abbreviated Scale of Intelligence were used to calculate the IQ scores. The Letter-Word ID, Word Attack, and Reading Fluency subtests of the Woodcock Johnson (WJ) Tests of Achievement were used to calculate the reading standard scores. Data are presented as means with standard deviations (SD) in parentheses, except where noted*.

Thirteen out of 38 children had to be removed from the analysis due to low accuracy. This issue does not appear unique to our cohort. For example, Castles et al. (1999) stated that “a number” of grade 2 children (mean age = 7 years, 10 months) needed to be removed from the analysis due to low accuracy, but did not state how many. Fifteen percent of the grade 3 children (mean age = 8 years, 6 months) tested in the Castles et al. (2007) study had to be removed due to low accuracy. The neighborhood size of stimuli used in the present study may explain why this experiment was more challenging than previous studies. Low N words and high N non-words are the most challenging stimuli to correctly classify. The low N word targets in the Castles et al. (1999) study had a mean N of 1.3, whereas in the present study they had 0 neighbors. The high N non-words in the Castles et al. (1999) study had a mean N of 8.9, whereas in the present study they had a mean N of 12.7 (range: 10–19).

### Design and stimulus materials

#### Design

The lexical decision task contained both word and non-word stimuli; only words were analyzed. The words and non-words varied by Orthographic Neighborhood Size (high, low) and Prime Type (repetition, form, unrelated). An additional Orthographic Neighborhood Size condition (matched N) was presented to adult participants as a control condition. Children and adolescents completed a neighbor knowledge test to measure their effective Ns. Adolescents also completed psychometric testing; children and adults did not, but some psychometric data were available from previous studies in the lab. We describe three discrete age groups in the Methods section, as the testing procedures were slightly different for each age group. However, in the analyses, age is treated as a continuous variable.

#### Stimulus materials: lexical decision task

Stimuli were white letter strings displayed in the center of the screen in Courier font on a black background. Stimuli subtended 0.57 visual degrees vertically and up to 1.64 visual degrees horizontally. The mask had a contrast value of 0.47 and the other stimuli had similar contrast values.

Target items were 210 4–5 letter English words and 210 4–5 letter legal non-words compiled using the e-Lexicon database (Balota et al., 2007). All the word targets shown to children and adolescents had a 3rd grade frequency ≥ 1 (Zeno et al., 1995). The target words had one of three orthographic neighborhood sizes: 70 were high N (10–19 orthographic neighbors), 70 were low N (0 orthographic neighbors), and 70 were matched/medium N (8–9 orthographic neighbors) (Balota et al., 2007). The target non-words had the same characteristics, save that the low N non-words had 1 orthographic neighbor. The orthographic neighborhood size for the matched N stimuli, shown to adults as a control for children's smaller vocabularies, was selected to approximate the expected effective N of the high N list for the youngest children. Specifically, stimuli in matched N list had 8–9 orthographic neighbors, which corresponded to the average number of neighbors per high N word target with a 3rd grade frequency ≥ 1 (Zeno et al., 1995).

Lexical properties of the target stimuli are displayed in Table 2. There were no effects of Orthographic Neighborhood Size or Lexicality on letter string length, and no effect of Orthographic Neighborhood Size on HAL (Hyperspace Analog to Language) frequency (Lund et al., 1995)^1^, HAL log frequency, and number of syllables for words, all *p*s > 0.10. The HAL frequency of the orthographic neighbors for the matched N words and high N words did not differ, *p* = 0.92. The 3rd grade frequency of the high N and low N words also did not differ, *p* = 0.39.

**Table 2:**
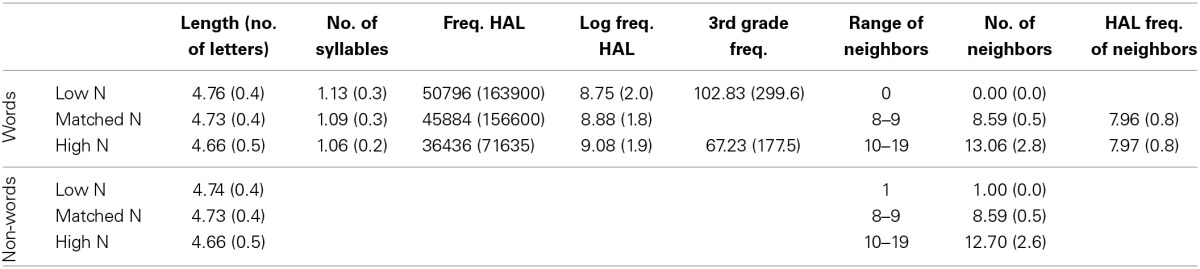
**Lexical properties of target stimuli**.

*Properties of the target stimuli expressed as mean (standard deviation). Frequency (freq.) measures were identified from the Hyperspace Analog to Language (HAL) estimates in the e-Lexicon database. Coltheart's N was used to identify neighbors*.

Each trial began with a forward mask consisting of a row of Xs, matched in length to the number of letters in both the prime and target (e.g., XXXX for a four-letter prime/target). Although an “x” is an English letter, it was only found in three targets. The forward mask was presented for 800 ms. Next, a prime was presented in lowercase font for 66.66 ms. This prime duration was chosen to closely approximate the prime duration in Castles et al. (1999) (57 ms) given our monitor refresh rate of 13.33 ms. Although the prime duration is slightly longer than usual, all of our participants except one reported either being completely unaware of the prime or of simply seeing a flicker on the monitor. Despite suggestions that prime visibility has a minimal impact on behavioral effects (Schmidt, 2013), we elected to exclude that participant because she occasionally made lexical decisions to the prime instead of the target stimulus. Then, a target was presented in uppercase font for 800 ms or until a lexical decision was made. Participants were instructed to determine whether the target was a real English word or a “made-up” word and to indicate their response by either the left or right button on a button box with the corresponding index finger. Response mappings were counterbalanced across participants.

The number of letters in the prime and target was equal for each trial. Repetition primes were characterized by the same item appearing in lowercase font as a prime and uppercase font as a target. Form primes were characterized as differing by one letter position from the target. All letter positions were changed an equal number of times. Unrelated primes shared a maximum of one letter in the same position as the target. The lexicality of the prime was selected to give no indication of the lexicality of the target item. For repetition prime trials, word primes always preceded word targets (e.g., rice-RICE) and non-word primes always preceded non-word targets (e.g., deat-DEAT). For form prime trials, non-word primes preceded word targets (e.g., ruce-RICE) and word primes preceded non-word targets (e.g., dean-DEAT). For unrelated prime trials, half of the targets were preceded by non-word primes and half were preceded by word primes (e.g., lunt-RICE or epic-RICE; tond-DEAT or milk-DEAT). All unrelated non-word primes were orthographically legal. Non-word form primes were created by replacing consonants with other consonants and vowels with other vowels. The prime lexicality was chosen to replicate the Castles et al. (1999) experimental design to facilitate cross-study comparisons.

For each target, three primes (repetition, form, and unrelated) were created. Initially, 12 lists (6 for adults, 6 for children/adolescents) were created with different combinations of the 3 prime types, so that every participant viewed each target once but, across the sample, every target was preceded by every prime type. Each list was then pseudorandomized with the constraint that no more than 6 examples of a particular response type were presented sequentially. Two pseudorandomized versions were generated for each list, yielding a total of 24 stimulus lists (12 for adults, 12 for children/adolescents) (see Supplementary Material for a list of stimuli).

#### Stimulus materials: neighbor knowledge test

The neighbor knowledge test served as our in-house vocabulary test and was used to measure the children's and adolescents' knowledge of the neighbors of the high N stimuli. Stimuli were white letter strings displayed in Courier font on a black background. Stimuli subtended 0.573 visual degrees vertically and up to 1.637 visual degrees horizontally. The possible targets were the 605 unique neighbors of the word targets from the lexical decision task. The foils were 195 orthographically legal non-words. Lexical properties of the target stimuli are displayed in Table 3. The targets and foils were randomly divided into 5 lists of 160 items. 20–30% (*M* = 24.38%) of each list consisted of non-word foils. Each list was pseudorandomized, with the constraint that no more than 6 examples of a particular response type were presented sequentially.

**Table 3:** **Lexical properties of neighbor knowledge test stimuli**.

	**Length**	**Freq. HAL**	**Log freq. HAL**	**Number of orthographic neighbors**	**Number of syllables**
Words	4.51 (0.5)	26640 (85148)	8.01 (2.2)	10.09 (4.2)	1.10 (0.3)
				range: 1–24	
Non-words	4.34 (0.5)			8.04 (2.7)	
				range: 0–22	

*Properties of the target stimuli expressed as mean (standard deviation). Abbreviations and conventions as in Table 2*.

Each trial began with a centered row of Xs presented for 800 ms, matched in length to the number of letters to the target/foil (e.g., XXXX for a four-letter item). Then, a centered target or foil was presented in lowercase font until the participant made a lexical decision. Response mappings were kept constant from the priming experiment.

Adults did not take the Neighbor Knowledge Test because we assumed that the adults knew most of the neighbors. The neighbors were fairly frequent [Hyperspace Analog of Language (HAL) mean: 26639.6, range: 16–1060831], and the adults were of high ability (estimated IQ = 126, all but one adult participant were students at Washington University in St. Louis).

### Procedure

Participants were tested individually in a dark, quiet, and windowless room. Stimulus presentation and response collection was controlled by PsyScope X (Carnegie Mellon University, Build 53) scripts running on an Apple OS X computer. Stimuli were displayed on a Trintron PC monitor. Participants' heads were held in place by a chin rest positioned 70 cm from the display monitor. Child and adolescent participants completed the practice trials, the lexical decision task, and the neighbor knowledge test. Adolescent participants also completed psychometric testing. Adult participants completed the practice trials and the lexical decision task.

Participants were instructed to determine whether each stimulus was a real word or a “made-up” word. Participants were told to respond as quickly and accurately as possible, and not to worry if they made an occasional mistake.

Additional instructions were given to children and adolescents to reduce the high false alarm rate seen during pilot testing. They were told that all of the words were fairly easy words, which they might have read before in books and whose meaning they knew. Additionally, if something looked like a word, but they hadn't read it before or did not know what it meant, it probably was a made-up word.

#### Practice trials

After receiving instructions, child and adolescent participants viewed 10 flashcards, with different example stimuli, and were asked to determine whether each stimulus was a word or a non-word. The experimenter gave feedback, pointing out that some of the non-words looked or sounded like real words. All participants were given 10 practice trials on the computer (with the same procedure as the lexical decision task trials).

#### Lexical decision task

Adults completed 420 experimental trials, with five breaks. Children and adolescents completed 280 experimental trials, with four breaks. The difference in the number of trials was due to adults also seeing the medium N stimuli. The task lasted approximately 20–30 min.

#### Neighbor knowledge test

Child and adolescent participants completed 160 experimental trials, with 1 break. The task lasted approximately 10 min. This task was always presented after the lexical decision task.

Participants were instructed that accuracy was more important than speed on this task. To reduce guessing, they were told to only answer “word” if they were sure that they had read the item before and knew what it meant.

#### Psychometric testing

Adolescent participants completed the Vocabulary and Matrix Reasoning subsets of the Wechsler Abbreviated Scale of Intelligence (WASI, Wechsler, 1999) and Letter-Word ID, Word Attack, and Reading Fluency subtests of the Woodcock Johnson (WJ) Tests of Achievement (WJ III-R COG; Woodcock et al., 2001). Although adults and children did not complete this testing, some of their IQ and reading ability measures (calculated using the same assessments) were available from prior studies. We believe that the subset is representative of the entire sample, as there was no systematic variation on the experimental task between the participants for whom scores were and were not available.

## Results

### Neighbor knowledge test

For child and adolescent participants, the effective N of the high N word stimuli was estimated from the neighbor knowledge test. Each child was not tested on every neighbor; estimates of each child's effective N were generated using the sample of neighbors on which he/she was tested. First, each neighbor word was weighted by the number of word targets (henceforth, “points”) from the lexical decision task for which it was a neighbor (e.g., “cases” was worth 5 points because it was a neighbor of 5 high N targets including “cages” and “bases”). A weighted estimate of the proportion of neighbors of the high N targets that each child or adolescent knew was computed to estimate their effective Ns (see Equation 1). First, we summed the points for each hit (i.e., each real word that the child identified as such). This sum was called the number of points earned. Then, we summed together the total possible points (# possible points). We multiplied the number of possible points by the false alarm rate to estimate the number of points the child earned through random guessing. We then subtracted this product from the number of points earned to calculate the number of points the child earned by knowing the vocabulary words, rather than by randomly guessing. We then divided this amount by the number of possible points to calculate the proportion of points the child earned by knowing the vocabulary words. This proportion was multiplied by the average N of the high N target words (13.06) to calculate the average number of neighbors that each child or adolescent knew. A weighted estimate was used because there was a great range in the number of targets (1–5) for which a given item was a neighbor. This weighted calculation allowed us to give more credit when known words were neighbors of multiple targets.

(1) $$\frac{\left(\#\text{ points earned}\right)-\left[\left(\text{false alarm rate}\right)*\left(\#\text{ possible points}\right)\right]}{\left(\#\text{ possible points}\right)}$$

On average, children knew 9.38 neighbors (*SD* = 1.03) of each target word and adolescents knew 9.98 neighbors (*SD* = 1.20) out of 13.06. Although this difference is small, the correlation with age was significant (*r* = 0.36, *p* = 0.01). Neighbor knowledge test scores strongly correlated with WASI raw vocabulary scores (*r* = 0.62, *p* < 0.01), but not WASI matrix reasoning raw scores (*r* = 0.30, *p* = 0.07)^2^, suggesting that the neighbor knowledge test tapped into an aspect of children's general vocabulary knowledge.

The neighbor knowledge test confirmed that target words were fairly high N for the children and adolescents. Furthermore, the “matched N” list (where *N* = 8.59) shown to the adults closely approximated, or slightly underestimated, the effective N for the children (i.e., 9.38). It is preferable for the matched N condition to slightly underestimate the average effective N for the children, because it is over-correcting for most children and very closely matching the effective N for the youngest children [the average effective N of the three youngest children (mean age = 8.95 years) was 8.65]. The matched N condition was therefore used in subsequent analyses as a control for differences in effective N across development by determining whether similar results were seen for adults with the matched N list and children and adolescents with the high N list.

### Lexical decision task

The adults, adolescents, and children were 93, 90, and 82% correct on all trials respectively.

We conducted mixed linear analyses using the lme4 package (Bates et al., 2010). The analysis methodology replicated that of Andrews and Lo (2012). First, we filtered the responses to examine only correct responses to word targets. Then we calculated the mean and standard deviation RT for each participant. Outlier RTs more than two standard deviations from a participant's mean were removed from the analysis (see Table 4). The negative inverse RTs were calculated as visual inspection showed that this best approximated a normal distribution and this transformation was used in similar studies (Andrews and Lo, 2012). The analyses treated participants and targets as crossed random effects. We assessed the effects of target neighborhood size and prime type with two orthogonal normalized contrasts comparing (a) average priming (mean of repetition and form primes as compared to unrelated primes) and (b) form and repetition primes. A generalized matrix inversion was then conducted on the contrast weights to yield interpretable main effects. To facilitate comparison with previous evidence of form priming, a second set of models tested generalized matrix inverted normalized contrasts that separately compared the form and repetitions primes with the unrelated primes. Higher order interactions of these contrasts with neighborhood size were included as fixed effects. Since the *t*-values obtained using linear mixed effects models are not conventionally associated with degrees of freedom, Markov-chain Monte Carlo simulations with 10,000 simulations were used to obtain the associated *p*-values.

**Table 4:** **Reaction time on lexical decision task**.

		**Children**	**Adolescents**	**Adults**
Low N	Repetition	799 (281)	649 (157)	551 (109)
	Form	836 (273)	677 (160)	582 (91)
	Unrelated	857 (244)	700 (165)	594 (91)
Matched N	Repetition	–	–	543 (97)
	Form	–	–	583 (97)
	Unrelated	–	–	584 (89)
High N	Repetition	799 (258)	626 (153)	535 (94)
	Form	822 (259)	672 (166)	582 (93)
	Unrelated	836 (268)	665 (144)	577 (89)

*The trim reaction time (ms) to correct word targets displayed as mean (standard deviation)*.

**Figure 1:**
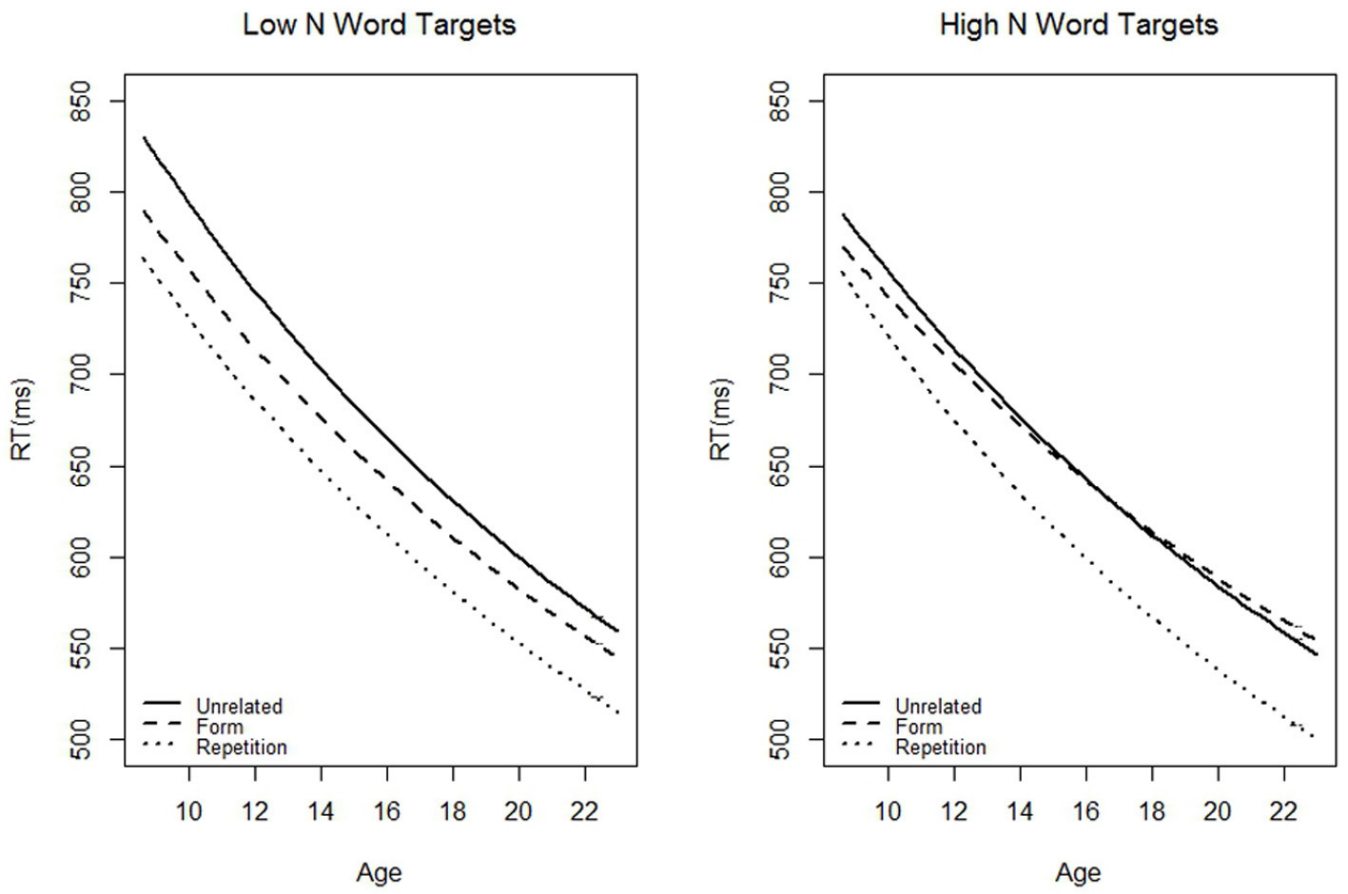
**The predicted (based on our models) average reaction time to targets preceded by the three different prime types as a function of age (in years)**. This model used the high N targets in adults.

We were interested in testing the three-way interaction between target orthographic neighborhood size (categorical high/low), participant age (continuous), and prime type (using the contrasts described above). The most straightforward support of our main hypothesis would be a significant interaction between age, neighborhood size, and the contrast between form and unrelated primes. This finding would suggest that the extent of form priming (as compared against the unrelated baseline, the typical calculation) to high N words changes with age. However, our hypothesis would also be supported if there were a significant interaction between age, neighborhood size, and the contrast between form and repetition primes. Repetition and form primes only differ by one letter. The significant interaction would suggest that the way participants respond to partially and fully matching primes preceding high N targets changes with age. We also regressed out factors which can affect reaction time: frequency, length, number of syllables, bigram mean, RT on the preceding trial, and accuracy on the preceding trial (see Table 5 and Figure 1). As one can see from the figure, RT decreased with age. For Low N targets, the effects of the three prime conditions were relatively constant across age: repetition primes were more beneficial than form primes which were more beneficial than unrelated primes. However, for high N targets, the effects of the three priming conditions varied with age. Although repetition primes were always the most beneficial, the benefit derived from repetition primes (as compared to unrelated primes) increased with age. In contrast, the benefit derived from form primes (as compared to unrelated primes) decreased with age. In fact, for the oldest participants, form primes had a slight inhibitory effect.

**Table 5:** **The coefficients and their significances in the model using the high N targets in adults**.

	***B***	**Std. Error**	***t***	***pMCMC***
Intercept	−1.623	0.0189	−85.62	0.0001
Log freq.	−0.0218	0.0033	−6.62	0.0001
Prev. RT	0.1225	0.0084	14.54	0.0001
Bigram mean	−0.0001	0.0001	−1.42	0.1502
Length	0.0179	0.0145	1.24	0.1996
No. of syllables	0.0395	0.0222	1.78	0.0684
Prev. accuracy	0.0431	0.0088	4.90	0.0001
Age	−0.0409	0.0040	−10.17	0.0001
N	−0.0287	0.0124	−2.31	0.0160
Age^*^N	−0.0010	0.0011	0.87	0.4120
**UNRELATED/REPETITION & FORM CONTRAST; FORM/REPETITION CONTRAST**				
U/R&F	−0.0759	0.0055	−13.90	0.0001
F/R	−0.0984	0.0062	−15.77	0.0001
Age^*^ U/R&F	−0.0017	0.0012	−1.35	0.1866
Age^*^ F/R	−0.0081	0.0014	−5.81	0.0001
N^*^ U/R&F	−0.0333	0.0109	3.05	0.0018
N^*^ F/R	−0.0414	0.0125	−3.31	0.0008
Age^*^N^*^ U/R&F	−0.0010	0.0024	−0.42	0.6706
Age^*^N^*^F/R	−0.0074	0.0028	−2.65	0.0112
**UNRELATED/FORM CONTRAST; UNRELATED/REPETITION CONTRAST**				
U/F	−0.0267	0.0063	−4.22	0.0001
U/R	−0.1251	0.0063	−20.02	0.0001
Age^*^U/F	0.0024	0.0014	1.70	0.0952
Age^*^U/R	−0.0057	0.0014	−4.09	0.0001
N^*^U/F	0.0540	0.0127	4.26	0.0001
N^*^U/R	0.0127	0.0125	1.01	0.3096
Age^*^N^*^U/F	0.0027	0.0028	0.95	0.3548
Age^*^N^*^U/R	−0.0047	0.0028	−1.69	0.0924
**RANDOM EFFECTS**				
	**Variance**	**Stdev**.		
Target	0.0042	0.0650		
Ordering by participant	<0.0001	0.0003		
Participant	0.0226	0.1504		

*pMCMC is the p-value obtained using Markov-Chain Monte Carlo simulations. N is an abbreviation for orthographic neighborhood size. U, F, and R are abbreviations for unrelated, form, and repetition priming respectively. Therefore, U/F represents the contrast between the unrelated and repetition priming conditions and U/R & F represents the contrast between the unrelated condition and both the repetition and form priming conditions, etc. All continuous variables are centered. Previous accuracy is a categorical variable, with a correct response being the baseline. Orthographic neighborhood size is a categorical variable with 2 levels. A contrast code was used to compare orthographic neighborhood size*.

Log frequency, orthographic neighborhood size, accuracy on the previous trial, and age were negatively correlated with RT, whereas RT on the previous trial was positively correlated with RT. Consistent with our key hypothesis, the three-way interaction between age, neighborhood size, and the contrast between form and repetition priming was significant, *t* = −2.65, *pMCMC* = 0.01. In the low N condition, form and repetition priming decreased slightly with age; in the high N condition, form priming greatly decreased with age whereas repetition priming increased with age (Figure 1). This interaction can be further unpacked by examining the predicted values. For low N targets, the amount of benefit derived from both repetition and form primes preceding low N targets decreased by about 20 ms between the ages of 9 and 22. After controlling for confounding variables, a hypothetical 9 year old (corresponding to the average age of the three youngest participants) would yield a 63.79 ms repetition priming effect and a 38.58 ms form priming effect; whereas a hypothetical 22 year old would yield a 44.25 ms repetition priming effect and a 15.04 ms form priming effect. A different pattern of results emerges for high N targets. For children, priming is more beneficial in the low than high N condition. A hypothetical 9 year old would yield a 32.26 ms repetition priming effect and a 19.49 ms form priming effect. In adults, however, repetition priming is equally beneficial in the high N condition (45.54 ms). Furthermore, although form primes benefited children in the high N condition, they actually inhibited adults (−6.3 ms).

**Figure 2:**
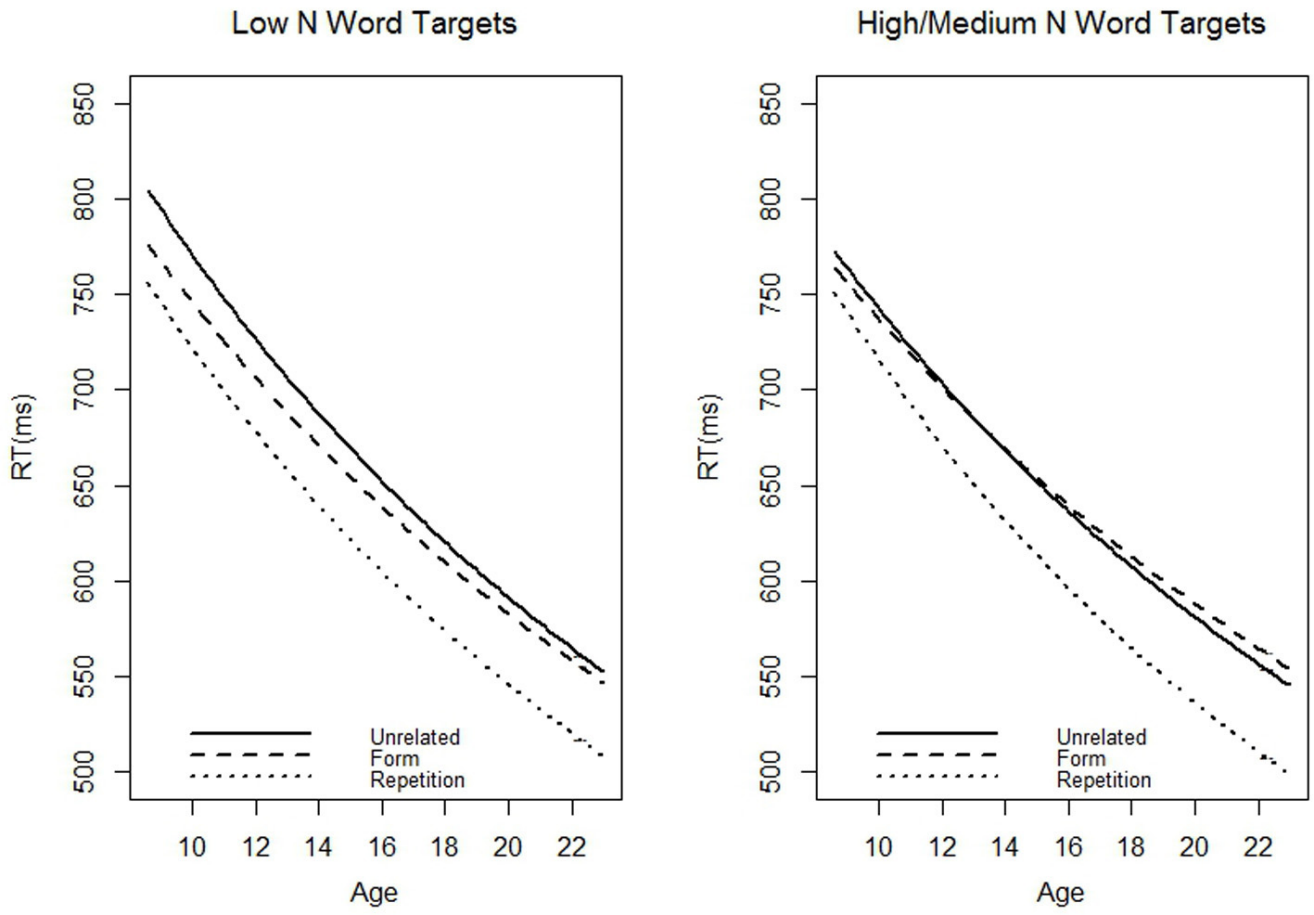
**The predicted (based on our models) average reaction time to targets preceded by the three different prime types as a function of age (in years)**. The trim method was used to clean outliers. This model used the medium N targets in adults. Note that the graph for the Low N words is slightly different than Figure 1 because the item-specific random effects are not included in this model.

We repeated the analysis using the matched N words for the adults. If the models using the high and matched N words were similar, it would suggest that development differences in priming effects are not solely due to vocabulary acquisition, as the effective N is controlled for in the matched N model. Target-specific random effects were excluded from matched N model since the adults and children/adolescents saw different items. The pattern of results seen in the matched N and high N models were very similar (see Table 6). All significant effects replicated, save that the three-way interaction between age, neighborhood size, and the contrast between form and repetition priming was a trend *t* = −1.78, *pMCMC* = 0.08. We re-ran the analyses with a slightly different method of cleaning outliers; replacing outliers with a boundary value rather than replacing them (fence method). Using this method of data cleaning, the three-way interaction between age, neighborhood size, and the contrast between form and repetition priming was significant, *t* = −2.33, *pMCMC* = 0.02. Although the interaction was only significant using one of the methods of cleaning outliers, it is important to remember that we over-corrected for effective N in this analysis. Therefore, we were able to find marginally significant effects even when the stimuli the adults saw had fewer neighbors than the children's effective N. Presumably, a closer matching of N would yield significant results. The coefficients for length, number of syllables, and bigram frequency were significant in the matched N model although they were not in the high N model, possibly due to the exclusion of target-specific random effects in the current model. The matched and high N model similarity can be discerned by comparing Figures 1, 2. Inspection of the predicted values reveals that even when the effective neighborhood size was matched, a hypothetical 22 year old adult showed more repetition priming (44.77 ms) and less form priming (−0.76 ms, again revealing slight inhibition) than the hypothetical 9 year old child did (repetition priming: 34.52 ms; form priming: 16.88 ms).

**Table 6:** **The coefficients and their significances in the model using the matched N targets in adults**.

	**Trim**				**Fence**			
	***B***	**Std. Error**	***t***	***pMCMC***	***B***	**Std. Error**	***t***	***pMCMC***
Intercept	−1.626	0.0184	−88.33	0.0001	−1.605	0.0184	−87.17	0.0001
Log Freq	−0.0230	0.0015	−15.53	0.0001	−0.0264	0.0016	−16.87	0.0001
Prev. RT	0.1172	0.0087	13.55	0.0001	0.1178	0.0088	13.37	0.0001
Bigram mean	−0.0001	<0.0001	−2.36	0.0210	0.0001	<0.0001	−2.39	0.0160
Length	0.0153	0.0063	2.41	0.0140	0.0203	0.0067	3.01	0.0026
No. of Syllables	0.0458	0.0095	4.84	0.0001	0.0523	0.0099	5.27	0.0001
Prev. Accuracy	0.0356	0.0089	3.98	0.0001	0.0505	0.0093	5.42	0.0001
Age	−0.0406	0.0041	−9.95	0.0001	−0.0406	0.0041	−9.96	0.0001
N	−0.0190	0.0054	−3.53	0.0004	−0.0156	0.0057	−2.74	0.0060
Age^*^N	0.0010	0.0012	0.81	0.4114	0.0021	0.0013	1.67	0.0918
**UNRELATED/REPETITION & FORM CONTRAST; FORM/REPETITION CONTRAST**								
U/R&F	−0.0771	0.0057	−13.62	0.0001	−0.0817	0.0060	−13.71	0.0001
F/R	−0.0926	0.0065	−14.30	0.0001	−0.0973	0.0068	−14.23	0.0001
Age^*^ U/R&F	−0.0018	0.0013	−1.39	0.1594	−0.0028	0.0013	−2.06	0.0380
Age^*^ F/R	−0.0073	0.0014	−5.02	0.0001	−0.0083	0.0015	−5.39	0.0001
N^*^ U/R&F	0.0260	0.0114	2.29	0.0214	0.0396	0.0119	3.32	0.0008
N^*^ F/R	−0.0295	0.0130	−2.27	0.0230	−0.0319	0.0137	−2.33	0.0180
Age^*^N^*^ U/R&F	−0.0015	0.0025	−0.58	0.5430	0.0011	0.0027	0.43	0.6694
Age^*^N^*^F/R	−0.0051	0.0029	−1.78	0.0754	−0.0071	0.0031	−2.33	0.0214
**UNRELATED/FORM CONTRAST; UNRELATED/REPETITION CONTRAST**								
U/F	−0.0308	0.0066	−4.69	0.0001	−0.0331	0.0069	−4.79	0.0001
U/R	−0.1234	0.0065	−19.05	0.0001	−0.1304	0.0068	−19.06	0.0001
Age^*^U/F	0.0019	0.0015	1.28	0.1946	0.0014	0.0015	0.89	0.3690
Age^*^U/R	−0.0054	0.0014	−3.72	0.0004	−0.0069	0.0015	−4.49	0.0001
N^*^U/F	0.0407	0.0131	3.10	0.0014	0.0556	0.0148	4.02	0.0001
N^*^U/R	0.0112	0.0130	0.86	0.3868	0.0237	0.0137	1.73	0.0838
Age^*^N^*^U/F	0.0011	0.0029	0.37	0.7296	0.0047	0.0031	1.52	0.1304
Age^*^N^*^U/R	−0.0040	0.0029	−1.39	0.1646	−0.0024	0.0031	−0.79	0.4270
**RANDOM EFFECTS**								
	**Variance**	**Stdev**.			**Variance**	**Stdev**.		
Ordering by participant	<0.0001	0.0003			<0.0001	0.0004		
Participant	0.0233	0.1526			0.0232	0.1524		

Abbreviations and conventions as in Table 5. Trim refers to the data cleaning method in which outliers are removed, whereas fence refers to the data cleaning method in which outliers are replaced with a boundary value.

**Figure 3:**
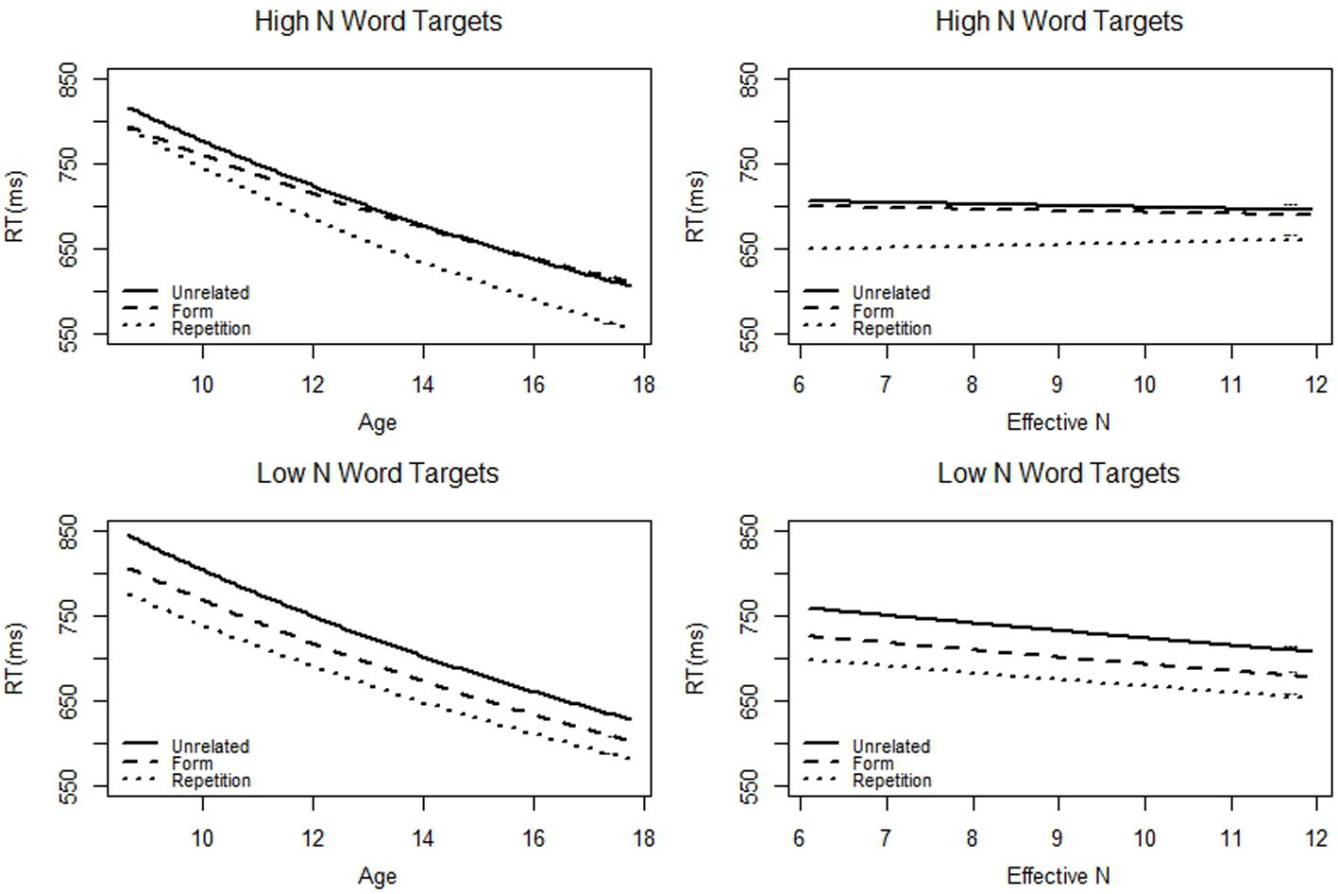
**The predicted (based on our models) average reaction time to targets preceded by the three different prime types as a function of age (in years) or effective N (in number of neighbors known) for just children and adolescents**. Effective N was calculated using the neighbor knowledge test.

Next, we tested whether the developmental trajectory was better explained by age or by the neighbor knowledge test. We restricted our test to children and adolescents because adults did not take the neighbor knowledge test. We used a linear mixed analysis, but instead of using Age as a factor in the three-way interaction, we used (Age + Neighbor Knowledge). This analysis was appropriate because the correlation between age and neighbor knowledge (*r* = 0.36) is well below accepted cutoffs for collinearity. Since the three way interaction was of main interest, we only ran the first pair of contrasts (unrelated/repetition&form; repetition/form). The results are displayed in Table 7 and Figure 3. In the interest of space, only factors involved in the three-way interaction are reported. The interaction between age, N, and the form/repetition priming contrast was significant, *t* = −2.50, *pMCMC* = 0.01. Furthermore, the interaction between Neighbor Knowledge, N, and the form/repetition priming contrast was non-significant, *t* = 0.59, *pMCMC* = 0.57. This analysis suggests that age, and not vocabulary, drives developmental differences in priming.

**Table 7:** **The coefficients and their significances in the model that tested the predictive power of effective N**.

	***B***	**Std. Error**	***t***	***pMCMC***
Intercept	−1.661	0.0440	−37.76	0.0001
Age	−0.0475	0.0098	−4.86	0.0001
Vocab	−0.0088	0.0224	−0.39	0.6426
N	−0.0428	0.0164	−2.60	0.0082
U/R&F	−0.0804	0.0124	−6.46	0.0001
F/R	−0.1029	0.0142	−7.23	0.0001
Age^*^N	−0.0032	0.0026	−1.20	0.2342
Vocab^*^N	0.0158	0.0060	2.62	0.0092
Age^*^ U/R&F	−0.0026	0.0028	−0.94	0.3528
Age^*^F/R	−0.0094	0.0032	−2.92	0.0046
Vocab^*^ U/R&F	0.0018	0.0064	0.29	0.7778
Vocab^*^F/R	0.0040	0.0074	0.54	0.6016
N^*^ U/R&F	0.0300	0.0250	1.20	0.2316
N^*^F/R	−0.0846	0.0285	−2.96	0.0032
Age^*^ N^*^U/R&F	0.0017	0.0056	−0.29	0.7774
Age^*^N^*^F/R	−0.0162	0.0065	−2.50	0.0102
Vocab^*^ N^*^U/R&F	0.0049	0.0129	0.38	0.6946
Vocab^*^N^*^F/R	0.0088	0.0149	0.59	0.5732
	**Variance**	**Stdev**.		
Target	0.0044	0.0662		
Ordering by participant	<0.0001	0.0004		
Participant	0.0262	0.1617		

*This model only included children and adolescents. Although not shown in the table, the length, frequency, bigram mean, and the number of syllables in the target were controlled for in the model, as was the participants' accuracy and RT on the preceding trial. Here, “vocab” refers to the effective N as calculated by the neighbor knowledge test. Abbreviations and conventions as in Table 5*.

## Discussion

Previous studies have shown that children are facilitated by both repetition and form primes preceding both low and high N targets. In contrast, adults do not show facilitation when form primes precede high N targets (Castles et al., 1999). However, it was unclear whether increases in written vocabulary size underlie these developmental changes. This study sought to replicate previous findings and test the hypothesis regarding vocabulary across a broader range of ages than had been previously studied. Our study replicated previous findings in that children were facilitated by repetition and form primes, but adults were facilitated in three conditions (high and low N repetition priming; low N form priming) but inhibited by form primes preceding high N targets. When we examined whether written vocabulary growth could explain this developmental differences, we found that it could not. Our models predicted developmental differences when controlling for effective N (using a matched N stimulus set). They also indicated that vocabulary size, measured using the neighbor knowledge test, could not predict priming effects.

Treating age as a continuous variable also allowed us to identify a previously unreported trend: the benefit derived from repetition primes preceding high N targets slightly increased over the course of development. Although previous developmental studies have not shown changes in repetition priming with age (Castles et al., 1999), there is evidence that more skilled adults (as measured by faster RTs in a lexical decision task) showed more repetition priming than low skilled adults (Kliegl et al., 2010). Since our adults responded much faster than our children, our results nicely dovetail with these findings.

Since written vocabulary does not seem to be related to developmental differences in priming, another mechanism must be at play. Andrews and Hersch (2010) identified a candidate mechanism: lexical precision. In an adult study, they found that spelling skill, but not written vocabulary size, was able to predict individual differences in masked form priming. Poor spellers were facilitated by form primes preceding high N words, whereas good spellers were slightly inhibited. Since spelling ability is a measure of orthographic precision, these results suggest that it is differences in lexical precision, rather than the number of neighbors known (i.e., written vocabulary size), which determine form priming effects. Although these effects were reported with adult participants, it is possible that a similar mechanism underlies developmental differences in priming. Children may show more facilitation due to form primes preceding high N targets because their orthographic representations are less precise.

Precise representations are fully specified so that a written word can fully determine the lexical representation to be activated, and this lexical representation can be quickly activated with minimal activation of its neighbors. Let us quickly summarize how an increase in the precision of lexical entries accounts for both our expected and rather surprising findings, before discussing the mechanism by which the lexical entry achieves this precision. The first finding is that adults derive equal benefit from repetition primes preceding both low and high N targets, whereas children derive more benefit in the low N condition. When a person with high quality lexical representations (presumably an adult) sees a repetition prime, the prime will quickly and correctly activate its corresponding lexical entry and nothing else. The correct activation of its lexical entry will make response time to the target faster. Therefore, adults will display equal repetition priming to low and high N targets. When a person with lower quality lexical representations (presumably a child) sees a repetition prime, it will activate its corresponding lexical representation and the lexical representations of its neighbors (if any). If the neighbors are slightly activated, they may weakly inhibit the correct lexical representation. Therefore, if the prime has no neighbors, the repetition prime will be more beneficial than if the prime has many neighbors. Therefore, children will display more repetition priming to low than high N targets.

This mechanism can also explain why children are facilitated by form primes preceding high N targets but adults are not. *Target preactivation* occurs because the prime and target share many of the same letters, so the letter level activates the target. This facilitation can be counteracted by *target neighbor suppression* due to lateral inhibition between orthographic neighbors at the word level. Thus, a form prime will activate all of its neighbors via the *target preactivation effect*. If the target has many neighbors, as in the high N condition, the target word and many of its neighbors will be activated. As people with high quality lexical representations are assumed to have more lateral inhibition, the target word would be strongly inhibited by its neighbors and the *target neighbor suppression* would override all facilitation from the *target preactivation effect*. In contrast, people with low quality lexical representations have less lateral inhibition, so the *target preactivation effect* would remain stronger than the *target neighbor suppression*. Note that in both cases, vocabulary is equated: people with low and high quality lexical representations know the same number of neighbors of a given target word. But, the lateral inhibition from a given neighbor is stronger in people with high quality lexical representations. Of course, the above argument is purely speculative as we, unfortunately, did not acquire measures of spelling ability (i.e., lexical precision). Nonetheless, the present results do not support the written vocabulary hypothesis. The strongest alternative explanation is that changes in lexical precision underlie developmental differences in form priming. Reading experience cannot explain the differential priming effects in adults with varying spelling abilities, because Andrews and Hersch (2010) found an effect of spelling ability while controlling for reading experience. However, the strength of reading experience as a predictive variable could be moderated by age, specifically, it may wane over development. It is possible that reading experience could be responsible for the results found in this study for children. Alternatively, writing experience, where children have to not only recognize, but also produce, the correct spelling could underlie these developmental changes. Future studies which directly correlate spelling ability and priming across the developmental spectrum are needed.

Before concluding, we acknowledge additional limitations of the present study. We restricted our target word stimuli to higher frequency, shorter words. It is unknown whether neighborhood effects on the development of form priming would persist across different word types. Second, to allow for a close comparison to previous developmental studies, we approximated as closely as possible the experimental timing used by Castles et al. (1999). However, adult form priming appears sensitive to subtle variations in experimental timing (Ferrand and Grainger, 1994). It is unknown if children display more adult-like patterns at longer prime durations. An additional concern is that the lexical decision task elicited large developmental differences in response time. Prior studies have demonstrated that apparent developmental differences in letter processing are reaction time dependent (Lachmann and van Leeuwen, 2008). However, after accounting for RT in a mixed linear effects model, age was still a significant predictor (*t* = −3.21, *pMCMC* < 0.01). In addition, it is unlikely that the developmental differences in RT reflect a difference in speed/accuracy trade-offs across age, as the children were both slower and more inaccurate than the adults.

Our results suggest that age-related factors beyond written vocabulary size underlie the developmental differences in high N form priming. Future studies may benefit from using designs that more closely match children's effective N and examining other individual differences (e.g., spelling ability) to pinpoint specific mechanisms that lead to developmental changes in the precision of lexical representations. Understanding why children are differentially affected by orthographic neighborhood size is crucial to understanding how children learn to distinguish between words with similar spellings, and why some children are not able to do so even after adequate instruction.

## Authors' note

The present study was supported by a grant from the National Institute of Child Health and Human Development [HD057076] to Bradley L. Schlaggar. We thank Rebecca Coalson, Alecia Vogel, Katie Ihnen, and Jessica Church for providing neuropsychological data on adult and child participants, Steve Petersen, Dave Balota, Alecia Vogel, Katie Ihnen, Elizabeth Votruba-Drzal, Charles Perfetti, Sally Andrews, and Jessica Church for helpful discussion, Fran Miezen for his help programming the task, Sally Andrews and Steson Lo for providing their code, and Nora Presson for assisting with R script. Portions of the data were presented at the Midwest Undergraduate Cognitive Science Conference. This research served as partial fulfillment of the requirements of the honors program in Biology at Washington University in St. Louis for Adeetee Bhide.

### Conflict of interest statement

The authors declare that the research was conducted in the absence of any commercial or financial relationships that could be construed as a potential conflict of interest.

## Footnotes

^1^The HAL frequency was derived using a corpus of words from Usenet, which includes all newsgroups using English dialog.

^2^These correlations are based on the data from all of the adolescents and 13 children. The scores from the 13 children included in the correlation analyses were obtained within a year of participation in the present study.
