# Supplementary material for: Developmental differences in masked form priming are not driven by vocabulary growth
Source: Front Psychol. 2014 Jul 11;5:667. doi: 10.3389/fpsyg.2014.00667 (PMC4093752; doi:10.3389/fpsyg.2014.00667)
Supplement: Supplementary file 1 [file Presentation1.PDF]

Appendix A

Stimuli for Masked Priming Paradigm

Word Targets				Nonword Targets			
Target	Identity Prime	Form Prime	Unrelated Prime	Target	Identity Prime	Form Prime	Unrelated Prime
High N Targets							
back	back	dack	fled	beal	beal	beak	soap
bake	bake	baku	jult	betch	betch	bench	larnt
balls	balls	bylls	dower	bines	bines	bikes	round
bases	bases	beses	pumor	bolly	bolly	holly	larnt
bear	bear	mear	curt	bure	bure	bore	whip
beats	beats	beits	spilm	cail	cail	coil	smot
bells	bells	belns	caged	cails	cails	calls	slave
best	best	bost	torf	cales	cales	cares	lornt
boots	boots	boops	marry	calt	calt	colt	whip
cages	cages	cagef	nadit	cates	cates	cater	spate
cakes	cakes	caces	musty	ceal	ceal	coal	ease
came	came	dame	durt	cines	cines	cites	kolan
cares	cares	yares	holly	coles	coles	codes	graze
cars	cars	carg	pect	cose	cose	cost	sart
cases	cases	cuses	stank	dake	dake	dare	milk
caves	caves	cuves	drent	dases	dases	cases	molat
comes	comes	fomes	grace	deat	deat	dean	milk
cords	cords	comds	gupid	dills	dills	fills	morat
deal	deal	deol	clad	dowed	dowed	dower	eight
dives	dives	duves	slipe	fower	fower	cower	lanim
done	done	dona	flag	gake	gake	bake	cups
fake	fake	faka	crit	gakes	gakes	games	tinal
fills	fills	finls	venal	graps	graps	grape	sound
halls	halls	jalls	nushy	hases	hases	hates	tinal
hears	hears	cears	aglow	hile	hile	hole	pray
lake	lake	lakr	spiq	hiles	hiles	hills	crute
lines	lines	lenes	aglow	hoves	hoves	hovel	glade
lives	lives	laves	greab	jines	jines	pines	crute
lock	lock	qock	acts	kails	kails	nails	glade
lower	lower	loter	adore	lages	lages	lager	crute
makes	makes	majes	nealt	lails	lails	fails	porch
mall	mall	mell	acre	lales	lales	lakes	snesk
miles	miles	milos	jarnt	lanks	lanks	lanky	porch
mines	mines	minos	acorn	lave	lave	love	chet
must	must	mumt	tiff	leat	leat	leak	paid
pack	pack	pacr	gunt	leats	leats	feats	shike
pears	pears	pearh	marsh	linds	linds	lines	purse
pets	pets	petp	lilm	mear	mear	mean	fard
piles	piles	hiles	elope	mears	mears	gears	light
pines	pines	kines	softi	mell	mell	melt	fard
rails	rails	lails	burnt	pakes	pakes	makes	merry
rake	rake	roke	sher	palt	palt	pelt	trad
rice	rice	ruce	epic	pank	pank	pant	cues

ride	ride	rade	snit	lans	lans	land	sher
rope	rope	rore	gawk	papes	papes	paper	grove
roses	roses	rones	snish	pares	pares	pared	smint
sails	sails	saols	given	peats	peats	pears	greed
seat	seat	seag	tump	pells	pells	bells	flart
seeds	seeds	seegs	tuned	pess	pess	pass	shoe
shake	shake	shabe	croag	poss	poss	post	bime
share	share	dhare	facts	rales	rales	rates	hound
shave	shave	chave	bourt	rines	rines	runes	flart
shore	shore	shori	braid	rits	rits	rats	shoe
sink	sink	senk	cril	rives	rives	river	dalor
spare	spare	sbare	minor	rolly	rolly	rolls	glare
stake	stake	ctake	tepek	rones	rones	ropes	ditch
stare	stare	sture	excel	sates	sates	sages	brame
store	store	stire	frath	saze	saze	size	fuss
takes	takes	tukes	pilot	seans	seans	seals	foltz
tales	tales	talis	spich	sind	sind	sand	lort
tall	tall	talp	axis	slank	slank	slant	fodes
tears	tears	teurs	glind	solly	solly	folly	litch
tiles	tiles	tilen	motor	sone	sone	sane	task
tower	tower	jower	skint	taked	taked	taken	crune
vines	vines	vinas	guise	tates	tates	tapes	chimp
waked	waked	yaked	smurk	tolly	tolly	tolls	night
wart	wart	wert	feud	warks	warks	warns	brown
waved	waved	wavew	amuse	wates	wates	water	crand
wears	wears	wuars	shilk	wend	wend	wind	plot
went	went	wend	sume	wores	wores	cores	spute
			Low N Targets				
asks	asks	usks	brat	abhar	abhar	abhor	mildo
buyer	buyer	buyen	chine	adbum	adbum	album	haste
chalk	chalk	chulk	today	adok	adok	amok	flup
churn	churn	ckurn	spelb	afarm	afarm	alarm	ducks
chute	chute	thute	toxin	afoub	afoub	afoul	crape
cliff	cliff	clisf	shuke	aigy	aigy	airy	doom
crumb	crumb	cramb	enact	ajir	ajir	ajar	spak
debts	debts	dibts	alupt	altep	altep	alter	horse
dwarf	dwarf	dwart	toxin	atgae	atgae	algae	rintu
earth	earth	earch	toxin	atre	atre	acre	wool
echo	echo	eclo	spav	axiab	axiab	axial	cough
eggs	eggs	aggs	tirl	befip	befip	befit	drawn
ends	ends	onds	tirt	beiga	beiga	beige	spoot
envy	envy	enva	girl	bisan	bisan	bison	loner
evil	evil	ekil	clay	bloff	bloff	bluff	spunt
exam	exam	esam	spil	bluos	bluos	blues	prime
faith	faith	gaith	token	cagus	cagus	cages	droil
ferns	ferns	fexns	cheln	crava	crava	crave	taste
first	first	finst	token	diapy	diapy	diary	brink
frogs	frogs	fwogs	timid	dimos	dimos	dimes	flune
froze	froze	frote	slamp	doap	doap	soap	rult
fruit	fruit	fruat	acres	dreet	dreet	creek	locks

geese	geese	geuse	slamp	eicht	eicht	eight	creen
ghost	ghost	ghosl	mason	elho	elho	echo	lips
girls	girls	girlj	slamp	excul	excul	excel	krids
glued	glued	gluid	emote	feght	feght	fight	cried
gourd	gourd	hourd	riger	flyem	flyem	flyer	groon
gruff	gruff	druff	emote	fuble	fuble	fable	plank
guard	guard	guarv	snime	gabla	gabla	gable	smorn
hawks	hawks	huwks	three	gerby	gerby	derby	lunch
hymn	hymn	hamn	kopt	havic	havic	havoc	jerun
idea	idea	udea	acid	hyrup	hyrup	syrup	cures
jazz	jazz	jawz	kopt	igeal	igeal	ideal	moona
jewel	jewel	jehel	tooth	knag	knag	snag	veer
knelt	knelt	khelt	elite	lanjo	lanjo	banjo	wooba
knife	knife	knifu	tusks	lemar	lemar	lemur	right
laugh	laugh	laush	tramp	limno	limno	limbo	crute
liar	liar	lior	clon	lunur	lunur	lunar	stove
midst	midst	hidst	adorn	mimoc	mimoc	mimic	vulta
mourn	mourn	mourt	judda	myshs	myshs	myths	funny
ninth	ninth	ninsh	tiple	nidy	nidy	tidy	drin
nouns	nouns	nauns	egert	nylen	nylen	nylon	crack
obey	obey	ebey	cows	oasy	oasy	easy	gord
odor	odor	odom	doap	odar	odar	odor	lord
okay	okay	okuy	cows	ofoe	ofoe	oboe	hogs
once	once	opce	doap	ofra	ofra	okra	path
proud	proud	groud	enemy	ojive	ojive	olive	hearf
quake	quake	quare	ferst	operi	operi	opera	later
rafts	rafts	ramts	niece	polki	polki	polka	queel
raise	raise	reise	antla	pugle	pugle	bugle	colds
ranch	ranch	ranck	fifty	purso	purso	purse	drash
range	range	ranfe	antla	quein	quein	quiet	foods
rinse	rinse	ripse	twigs	quop	quop	quip	lapy
seize	seize	deize	antla	revol	revol	revel	jumps
swarm	swarm	swark	plint	rical	rical	rival	faner
their	their	thear	jonki	rowir	rowir	rower	peach
third	third	tjird	nasal	sless	sless	bless	lotch
trout	trout	lrout	jonki	slond	slond	blond	timer
truth	truth	trutf	favor	slonk	slonk	slink	truim
tufts	tufts	tusts	jonki	spocy	spocy	spicy	aired
twist	twist	thist	favor	swens	swens	swans	yarty
ugly	ugly	igly	menu	tarve	tarve	carve	couch
urge	urge	urgo	pash	ucly	ucly	ugly	frot
views	views	viaws	mount	ugna	ugna	ulna	corp
voice	voice	vuice	shewn	vaggy	vaggy	baggy	sleer
wharf	wharf	sharf	tutor	vuft	vuft	tuft	coin
wheel	wheel	wheul	fuben	wedgo	wedgo	wedge	gramb
which	which	whech	tutor	whilo	whilo	while	forks
width	width	wadth	fuben	yeor	yeor	year	ents
young	young	yound	unify	zaly	zaly	zany	krut
Matched N Targets							
bands	bands	cands	eject	beels	beels	heels	spank

batch	batch	banch	piyab	blace	blace	black	muner
beads	beads	beadx	eject	blate	blate	blade	minus
bond	bond	bund	kawp	boves	boves	loves	flant
bores	bores	boras	eject	brap	brap	brag	hues
burn	burn	curn	also	caker	caker	cakes	shode
cooks	cooks	coiks	eapet	citer	citer	cider	grows
cost	cost	cosg	hike	clows	clows	claws	prent
cower	cower	ciwer	eldan	crips	crips	drips	diner
crown	crown	croln	enjoy	curp	curp	curb	flib
dark	dark	tark	shiv	dacks	dacks	docks	toner
dean	dean	deun	navy	datch	datch	batch	crend
deep	deep	deek	shiv	dites	dites	dotes	spure
doses	doses	dises	mayor	dooks	dooks	books	clute
fares	fares	faras	thide	dows	dows	cows	weld
fates	fates	qates	mayor	fally	fally	folly	wired
fears	fears	feors	bivel	faunt	faunt	fauna	hitch
fires	fires	firet	mayor	flass	flass	flask	proot
gases	gases	guses	bivel	foed	foed	feed	grab
grade	grade	grake	bluff	fope	fope	rope	hulk
grape	grape	trape	pafta	foses	foses	fuses	mantly
grass	grass	gress	bluff	fulls	fulls	fills	creed
grate	grate	gratu	egess	fushy	fushy	fussy	plont
grown	grown	gpown	bluff	gaked	gaked	raked	dusty
hates	hates	hatis	tearp	gaws	gaws	gawk	jell
hides	hides	lides	braid	gilly	gilly	gills	trick
kind	kind	kild	easy	gound	gound	pound	tatch
laid	laid	laiv	deat	grake	grake	brake	water
leads	leads	liads	mango	grame	grame	grime	smoit
lever	lever	leveb	skash	herp	herp	harp	lobe
life	life	yife	unit	jold	jold	jolt	ferb
lift	lift	liht	pume	jower	jower	cower	dandy
lined	lined	qined	towns	laper	laper	laser	prouk
loves	loves	laves	teaky	loas	loas	loan	yarn
match	match	matsh	frenk	lonk	lonk	link	parn
mean	mean	muan	nofe	lusk	lusk	lust	maid
meats	meats	meuts	topic	mabs	mabs	maps	drue
mills	mills	millp	entap	miled	miled	miles	looks
move	move	moge	edit	natch	natch	notch	plort
nails	nails	naiws	fibel	nates	nates	names	tight
null	null	rull	edit	nive	nive	nine	kulo
packs	packs	pacds	toren	nust	nust	nest	glue
peel	peel	peeg	exit	nusty	nusty	rusty	speen
pour	pour	paur	neel	parm	parm	perm	lied
races	races	racef	broth	pents	pents	pants	jodra
rages	rages	vages	bilan	pives	pives	pines	leard
rains	rains	raens	broth	podes	podes	poles	zulun
raked	raked	daked	bilan	poots	poots	pouts	train
reach	reach	riach	float	pord	pord	pork	liks
scare	scare	scabe	soral	raded	raded	faded	peals
seals	seals	sials	float	rees	rees	reel	glip

shack	shack	sheck	prike	romes	romes	roles	singe
shop	shop	shor	tuna	roods	roods	rooms	rumte
slips	slips	slups	phort	roop	roop	root	shin
slots	slots	slors	trunk	rosed	rosed	roses	brant
some	some	pome	tisk	sarry	sarry	sorry	bodes
spake	spake	spawe	exult	shase	shase	shake	norts
spine	spine	spina	froat	sheed	sheed	speed	fully
stone	stone	swone	fault	shunk	shunk	shunt	plart
tails	tails	tailj	smake	slade	slade	slate	pitch
tapes	tapes	sapes	femur	slite	slite	slits	tarop
tents	tents	tects	smake	slock	slock	slack	liter
time	time	pime	wand	storn	storn	stork	crult
wager	wager	wuger	duval	stort	stort	storm	taper
waves	waves	wavis	month	tham	tham	them	jild
wells	wells	wolls	jimit	tined	tined	timed	folds
wires	wires	wihes	month	tound	tound	hound	sweet
wives	wives	wivey	tutor	trice	trice	trick	bleen
words	words	wonds	duval	vides	vides	video	pasty
zone	zone	zote	fern	vills	vills	villa	shpun
